# Triglyceride-Rich Lipoproteins and Remnants: Targets for Therapy?

**DOI:** 10.1007/s11886-016-0745-6

**Published:** 2016-05-23

**Authors:** Geesje M. Dallinga-Thie, Jeffrey Kroon, Jan Borén, M. John Chapman

**Affiliations:** Department of Vascular Medicine, Academic Medical Center, Amsterdam, The Netherlands; Department of Experimental Vascular Medicine, Academic Medical Center, Amsterdam, The Netherlands; Department of Molecular and Clinical Medicine, University of Gothenburg and Sahlgrenska University Hospital, Gothenburg, Sweden; INSERM and University of Pierre and Marie Curie, Pitie-Salpetriere University Hospital, 75651 Paris Cedex 13, France

**Keywords:** Triglycerides, Apolipoprotein, Heparin sulphate proteoglycan

## Abstract

It is now evident that elevated circulating levels of triglycerides in the non-fasting state, a marker for triglyceride (TG)-rich remnant particles, are associated with increased risk of premature cardiovascular disease (CVD). Recent findings from basic and clinical studies have begun to elucidate the mechanisms that contribute to the atherogenicity of these apoB-containing particles. Here, we review current knowledge of the formation, intravascular remodelling and catabolism of TG-rich lipoproteins and highlight (i) the pivotal players involved in this process, including lipoprotein lipase, glycosylphosphatidylinositol HDL binding protein 1 (GPIHBP1), apolipoprotein (apo) C-II, apoC-III, angiopoietin-like protein (ANGPTL) 3, 4 and 8, apoA-V and cholesteryl ester transfer protein; (ii) key determinants of triglyceride (TG) levels and notably rates of production of very-low-density lipoprotein 1 (VLDL1) particles; and (iii) the mechanisms which underlie the atherogenicity of remnant particles. Finally, we emphasise the polygenic nature of moderate hypertriglyceridemia and briefly discuss modalities for its clinical management. Several new therapeutic strategies to attenuate hypertriglyceridemia have appeared recently, among which those targeted to apoC-III appear to hold considerable promise.

## Introduction

Whether plasma triglycerides, or more specifically, the lipoprotein particles in which they are transported between sites of absorption, lipolysis and remodelling and catabolism constitute an independent risk factor for CVD has been the subject of debate for decades [1]. Significant progress has been made of late in resolving this question as a result of three elements: firstly, the availability of prospective data focusing on the relationship between circulating TG levels and cardiovascular risk in large cohorts, secondly, observations made in the postprandial, non-fasting period, allowing analysis of this relationship over a substantially greater range of TG concentrations as compared to the fasting state and thirdly, analytical approaches which allow an estimation of the cholesterol burden carried in potentially atherogenic remnant particles [2–5, 6•]. Thus, accumulating evidence demonstrates a strong correlation between the risk of CVD and both non-fasting (postprandial) and fasting plasma TG levels. Furthermore, large prospective epidemiologic studies focused on non-fasting TG in response to normal food intake have demonstrated significant associations between increased CVD events with elevated concentrations of non-fasting TG [2, 3, 7]. Indeed, a meta-analysis of 17 prospective studies with 2900 CHD endpoints revealed that an increment of 1 mmol/L in fasting TG levels was associated with a 14 % increase in CVD risk [5]. However, this strong correlation is often lost or attenuated in multivariate analysis, principally as a consequence of the strong link between hypertriglyceridemia and other cardiovascular risk factors such as low high-density lipoprotein (HDL) cholesterol, obesity and insulin resistance [8, 9•]. Further support for a causative role of triglyceride-rich lipoproteins (TRLs) in CVD arises from genetic studies [4, 10–12]; such studies equally indicate that remnant particles, which represent the partially degraded products of TRLs (i.e. chylomicrons and very-low-density lipoprotein (VLDL)), play a key role in the pathophysiology of atherosclerotic vascular disease.

Hypertriglyceridemia is generally defined and diagnosed as fasting plasma TG > 1.7 mmol/L or >150 mg/dL and is the consequence of environmental, behavioural and genetic factors, among which lifestyle is prominent (alcohol use, smoking, a high carbohydrate diet and obesity) [13••]. Severe hypertriglyceridemia with plasma TG levels  > 10 mol/L (885 mg/dL) is typically of genetic origin, notably in the pediatric age group, and is associated with elevated risk of pancreatitis [14]. Further understanding of the pathobiology which underlies the atherogenicity of TRLs and their remnants will undoubtedly enable us to identify novel therapeutic targets; the translation of such targets into innovative therapeutic agents may significantly decrease cardiovascular risk in large numbers of hypertriglyceridemic individuals who currently remain at high risk despite optimal treatment according to current guideline recommendations.

### Production and Intravascular Metabolism of Triglyceride-Rich Lipoproteins and Remnants

Triglycerides represent the transport module for fatty acids which provide an essential source of energy upon oxidation in mitochondria. A major source of TG is derived from dietary fat consumption. Dietary TGs are transported in intestinally derived apolipoprotein (apo) B48-containing chylomicrons, which enter the systemic circulation through the lymphatic system and target the heart as the first organ for delivery of fatty acids to fulfil energy requirements. The liver plays a cental role in TG homeostasis and maintains a steady state between TG synthesis, secretion and oxidation. In contrast to adipose tissue, the liver does not serve as an organ for TG storage under normal physiologic conditions. The liver can take up fatty acids derived from lipolysis in adipose tissue or from circulating lipoproteins but may equally synthesise fatty acids from carbohydrates in the process of de novo lipogenesis. In the liver, fatty acids can be partly stored as TG in lipid droplets or oxidised to generate energy in mitochondria in the process of beta-oxidation or packaged in apo B100-containing VLDL particles and secreted into the systemic circulation where they serve as a source for energy for peripheral tissues. The molecular pathway involved in the packaging of TG into both chylomicron and VLDL particles is remarkably similar, involving microsomal triglyceride transfer protein (MTTP) as described in detail elsewhere [15, 16].

Triglycerides cannot pass through cell membranes freely. Consequently, intravascular lipolysis is an essential process for release of free fatty acids, which can then be taken up via specific fatty acid transporters or other yet unknown mechanisms. The underlying mechanism is still only partly understood, but identification of some of the key proteins involved has allowed progress in our understanding. Lipoprotein lipase (LPL) is the key enzyme which drives TG hydrolysis along the luminal surface of capillaries, whereas the recently identified protein glycosylphosphatidylinositol HDL binding protein 1 (GPIHBP1) provides the platform to allow lipolysis to occur at the endothelial cell surface [17, 18]. Lipoprotein lipase is synthesised in macrophages, adipocytes and myocytes and must be transferred to the luminal site of the endothelial cell to become active, a process which is facilitated by GPIHBP1 [18, 19]. Strict regulation of LPL production and activity is critical in different tissues and organs. LPL can be regulated at the level of transcription by both peroxisome proliferator-activated receptor (PPAR)α and PPARγ through binding to a PPRE element in the 5′ regulatory region of the LPL gene [20, 21]. PPARα is intimately involved in lipid metabolism in the liver, whereas PPARγ is more closely involved in adipose tissue lipid homeostasis thereby regulating LPL action in a tissue-specific manner. More recently, different microRNAs (miR29-a, 497b, 1277, 410) have been found to be involved in posttranslational regulation of LPL; the exact mechanism(s) has not as yet been elucidated [22, 23]. Both nutritional (fasting vs fed state) and hormonal status play central roles in the regulation of LPL expression in adipose tissue. In fed conditions, LPL activity is high due to the effect of insulin, thereby resulting in increased uptake of fatty acids. Interestingly, LPL is more strictly regulated in heart and skeletal muscle since these tissues need a continuous supply of fatty acids for energy production [24].

### Regulation of the Lipolytic Process

**Fig. 1:**
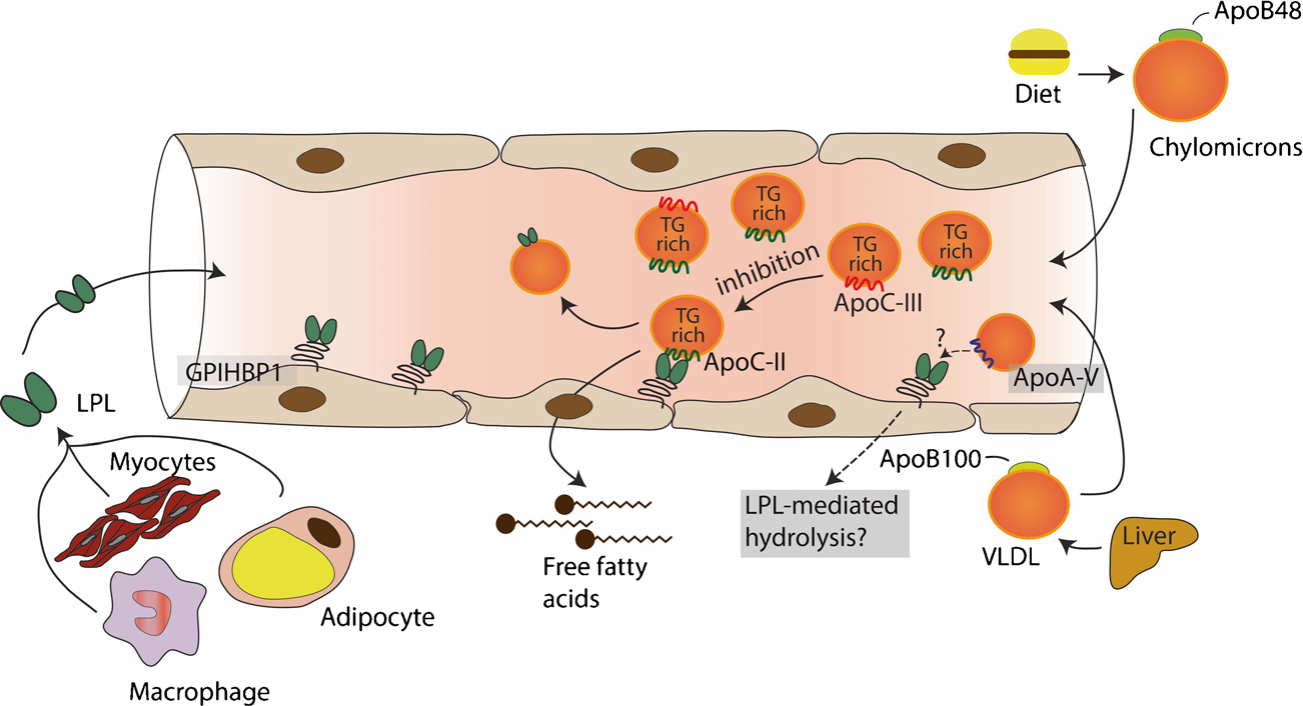
LPL is synthesised in parenchymal cells in muscle and adipose tissue and then transported to the endothelial cell surface. LPL-mediated TG lipolysis at this surface is the first essential step in TG homeostasis. TGs are hydrolysed by LPL bound to GPIHBP1 in a process that is dependent on apoC-II. ApoC-III and apoA-V are potential inhibitors of LPL-mediated lipolysis. Upon TG hydrolysis, free fatty acids are taken up by surrounding tissues

In vivo LPL action is regulated by several proteins including apolipoprotein (apo) C-II, apoA-V, apoC-III and angiopoietin-like protein (ANGPTL) 3, 4 and 8 [25] (Fig. 1). ApoC-II is a 79 amino acid peptide of hepatic origin containing a C-terminal domain involved in LPL activation and an N-terminal domain involved in lipid binding. Very little is known of the regulation of apoC-II synthesis. ApoC-II circulates in plasma on TG-rich lipoprotein particles as well as on HDL and is the rate-limiting protein required for normal LPL activity to occur. Patients with complete loss-of-function mutations in *APOC2* have severe hypertriglyceridemia similar to LPL deficiency [26]. On the other hand, increased plasma apoC-II levels are associated with increased plasma TG levels suggesting that a surplus may have an inhibitory effect on LPL function. ApoC-II may be important for guiding TG-rich lipoproteins to the active site of LPL at the endothelial cell surface [27, 28]. The proposed working model for apoC-II involves a mechanical process occurring during hydrolysis of lipoprotein TG and results in increased surface pressure with concomitant conformational change in apoC-II structure, followed by the release of an apoC-II-phosphatidyl choline complex to HDL [29]. The amino acid residues tyr63, ile66, asp69 and gln70 in the C-terminal helix of apoC-II are essential for LPL activation and have now been used to create an apoC-II mimetic peptide that promotes lipolysis on TG-rich lipoproteins by LPL and may represent a new therapeutic target [30, 31].

ApoC-III is a potent inhibitor of LPL function and is a 99 amino acid protein containing three sialic acid residues; it is synthesised mainly in the liver and to a small extent in the intestine. Different transcription factors may regulate *APOC3* gene expression such as PPARα and Fox01 [20, 32]. ApoC-III circulates on TG-rich lipoprotein particles as well as on HDL [33]. The apoC-III protein undergoes O-linked glycosylation by GALNT2 resulting in the presence of three isoforms: apoC-III 0, 1 and 2, which is impacting on apoC-III function [34]. Genetic studies have provided insight into the function of apoC-III. With respect to the potential relationship of circulating apoC-III levels to cardiovascular risk, a null mutation, p.R19X (rs56353203), was found to be associated with low plasma TG levels and attenuated subclinical atherosclerosis in the Amish population [35, 36]. Additional evidence was provided in a number of epidemiological studies showing the causal relationship between genetic variants in *APOC3*, plasma TG and CVD risk [37–39]. ApoC-III is now recognised as a multifaceted protein involved in different metabolic processes related to TG homeostasis. Firstly, apoC-III may inhibit hepatic clearance of TG-rich remnant particles by interfering with receptor binding sites [33]. Secondly, apoC-III has been recognised to inhibit LPL-mediated lipolysis in vitro; kinetic studies in human subjects do not however favour this concept [40]. Apparently, the ratio of apoC-III to apoC-II molecules on the surface of VLDL particles is the main determinant for LPL inhibition to occur. In vitro studies have shown that apoC-III/apoC-II ratios >5.0 are effective in inhibiting LPL action [41], a molar ratio which does not occur in human physiology. Finally, recent clinical trials in hypertriglyceridemic LPL-deficient patients using an allele-specific oligonucleotide (ASO) against apoC-III are in line with the concept that apoC-III has a major role in the hepatic uptake of remnant particles [42]. Thus, apoC-III emerges as an important drug target for reducing residual cardiovascular risk in hypertriglyceridemic subjects [43].

ApoA-V is a 366 amino acid protein primarily of hepatic origin. Circulating plasma apoA-V concentrations are very low which means that at most only 4 % of VLDL particles carry one apoA-V molecule [44]. Despite such low abundance, evidence supporting an essential role for apoA-V in TG metabolism is accumulating. Rare variants in or close to the *APOA5* gene locus are consistently associated with plasma TG levels and risk for CVD [45, 46]. However, plasma apoA-V levels are positively associated with plasma TG in humans, an observation which to date has not been fully understood [44, 47, 48]. Most studies on apoA-V function have been performed in mice overexpressing human apoA-V or in in vitro models using apoA-V liposomes [49]. In all of these models, apoA-V concentration is elevated in comparison to the physiological concentrations typically seen in humans. Moreover, the physiological context in which apoA-V is functional, i.e. in the presence of apoC-II, apoC-III or apoE on the same lipoprotein particle, and which all compete for similar functions, is missing. Interestingly, injection of apoA-V rHDL into *Apoa5*^*−/−*^ mice induces a rapid decline in plasma TG levels whereas a similar injection in *Gpihbp1*^*−/−*^ mice had no effect [50], suggesting that apoA-V might be involved in binding of TG-rich lipoproteins to GPIHBP1, thereby allowing LPL-mediated TG hydrolysis to proceed [51]. Whether apoA-V is involved in LPL-mediated TG lipolysis in humans has not yet been established.

ANGPTL3, 4 and 8 have been implicated in TG homeostasis. ANGPTL proteins contain a signal peptide, an N-terminal coiled-coil domain and a C-terminal fibrinogen-like domain [52]. ANGPTL4 is produced in adipose tissue, whereas ANGPTL3 is produced in the liver and ANGPTRL8 in both adipose tissue and the liver [53]. ANGPTL4 expression is increased under fasting conditions, whereas ANGPTL8 levels are increased in the fed state. ANGPTL3, 4 and 8 have been implicated in LPL action. In fasting conditions, in which ANGPTL4 expression in adipose tissue is upregulated, LPL action may be suppressed by ANGPTL4, resulting in rerouting of fatty acids towards other organs for supply of energy. ANGPTL4 effectively inhibits LPL action by converting the active dimer into an inactive monomer [54]. However, evidence has accumulated to show that ANGPTL4 could also act as a reversible, non-competitive inhibitor of LPL [55]. Such inhibition occurs solely when LPL is in a complex with ANGPTL4 and leads to restoration of LPL activity upon dissociation of the complex. Alternatively, ANGPTL4 may directly bind LPL that is bound to GPIHBP1 and in this manner inactivate the protein, whereafter dissociation from GPIHBP1 occurs [56]. Although the role of ANGPTL3 in regulation of lipolysis is less well understood, mutations in the *ANGPTL3* gene in humans are associated with reduced TG and cholesterol levels and elevated LPL activity [57]. ANGPTL3 is activated by proteolytic cleavage, leading to the release of the N-terminal domain, which has been shown to inhibit LPL and therefore result in reduced TG clearance. Recent data have emerged that ANGPTL8, also known as betatrophin or lipasin and a paralog of ANGPTL3, is able to interact with ANGPTL3, facilitating the cleavage of its N-terminal domain and thereby regulating its activity [58, 59]. In conclusion, all three ANGPTL isoforms are able to impact LPL activity and thereby lead to altered plasma TG levels.

### Determinants of Plasma Triglyceride Levels and Heterogeneity of Triglyceride-Rich Lipoprotein Particles

Very-low-density lipoprotein particles of hepatic origin can be subdivided on the basis of their size and role in TG metabolism; the larger, less dense particles are defined as VLDL1 (Sf 60–400) and the smaller as VLDL2 (Sf 20–60). Elevation of levels of large TG-rich VLDL1 is the major determinant of plasma TG concentrations in both normal and insulin-resistant individuals [60]. Increased plasma concentrations of VLDL1 can result either from hepatic oversecretion and/or impaired clearance of TRL remnants from the circulation [61, 62]. Hepatic oversecretion of VLDL1 particles is linked to increased liver fat and hyperglycemia [61, 63, 64]. Increased liver fat is equally associated with impaired suppression of VLDL1 secretion and results in oversecretion of VLDL1 particles [64, 65]. Recent findings in in vivo kinetic studies have shown that kinetic indices for VLDL1-TG catabolism are stronger determinants of circulating plasma TG concentration than kinetic parameters for the increased secretion of VLDL1 [66, 67•]. In particular, these studies revealed strong correlations between the catabolism of VLDL1-TG levels and the plasma concentration of apoC-III [66, 68]. The principal mechanism underlying the impairment of the catabolism of TRLs by apoC-III remains unclear, since apoC-III impairs both intravascular lipolysis by LPL and LPL-independent clearance of TRLs [69]. The significance of apoC-III in the hepatic clearance of TRL was recently illustrated by the markedly accelerated catabolism of TG-rich remnant particles in human subjects with deficiency of apoC-III [70].

Remnants are generated when chylomicrons and VLDL particles are remodelled during TG hydrolysis by LPL and are concomitantly enriched in cholesteryl esters by the action of the cholesteryl ester transfer protein (CETP). Thus, as TGs are removed, remnant particles become enriched with cholesteryl esters [71].

### Subendothelial Accumulation of Atherogenic Lipoproteins Induces Atherogenesis

**Fig. 2:**
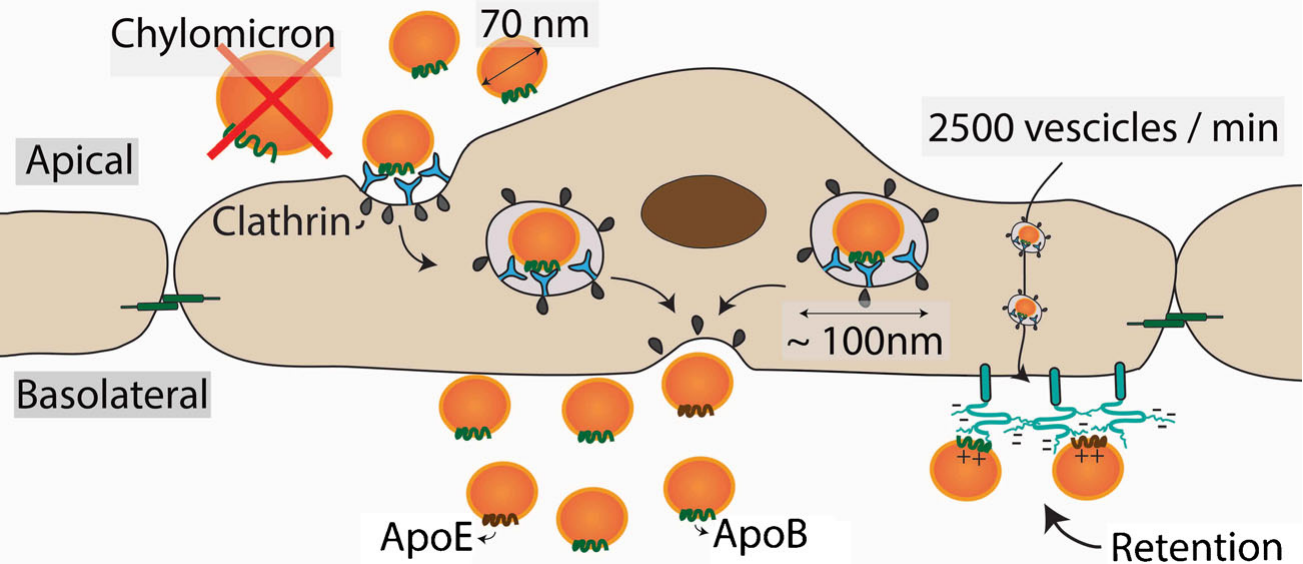
Transcytosis enable the influx of lipoproteins over the vessel wall. This process is mediated by clathrin. The average diameter of these transport vesicles are around 100 nm, which only allows transport of lipoproteins with the size of 70 nm or smaller, thereby excluding chylomicrons and large VLDL remnant particles. The average transport speed is around 2500 vesicles per minute. The retention of lipoproteins in the subendothelial space is mediated by the interaction between positively charged residues on apoB and apoE and the negatively charged sulphate groups in the glycosaminoglycans chains of HSPG expressed on the vessel wall

Lipoproteins in the circulation normally flux into and out of the arterial wall by transcytosis, a transport system in which lipoproteins and other macromolecules are transported across the endothelial cell in specialised clathrin-coated vesicles (Fig. 2) [72]. The transcytosis pathway has not previously attracted attention, but recent studies indicate that the process is responsive to LDL levels in the blood [73–75]. The transport vesicles are about 100 nm in diameter, and therefore, the transcytotic transport system is restricted to lipoproteins smaller than approximately 70 nm in diameter. Thus, larger lipoproteins, such as chylomicrons and large VLDL particles, cannot transverse the endothelium [76, 77]. This size limitation explains why individuals with lipoprotein disorders involving accumulation of large lipoproteins, such as chylomicrons in LPL-deficient patients, do not develop atherosclerosis. The capacity of the transcytotic transport system is very high; indeed, it has been estimated that about 2500 transport vesicles leave the plasma membrane every minute. Therefore, it is not the influx of lipoproteins into the artery wall that is rate limiting and thus determines the concentration of atherogenic lipoproteins in the artery wall but rather the selective subendothelial retention of lipoproteins in the artery wall [78]. Such retention is mediated by ionic interactions between positively charged residues in apoB and apoE on the atherogenic lipoproteins [79–81] and negatively charged sugar and sulphate groups in the glycosaminoglycan chains of the arterial wall proteoglycans.

### Postprandial Hypertriglyceridemia and Atherogenicity of Remnant Particles

For many years, accumulation of chylomicron and chylomicron remnants in plasma was believed to be the essential cause of postprandial hypertriglyceridemia [82, 83]. However, it is now established that although approximately 80 % of the increase in postprandial TG is due to chylomicrons [84], approximately 80 % of the increase in particle number is accounted for by VLDL particles [85, 86]. The underlying reason is that chylomicrons and VLDL particles are cleared from the circulation by common pathways and therefore compete for clearance [76], even if chylomicrons seem to be preferentially cleared [87]. Increased secretion of liver-derived VLDL is therefore causatively linked to postprandial accumulation of chylomicron remnants [87]. As discussed above, remnant particles contain significant amount of cholesteryl esters and can enter the arterial wall, even if their size results in attenuated transport across the endothelium as compared to smaller LDL particles. However, since each remnant particle contains approximately 40 times more cholesterol compared with LDL, elevated levels of remnants may lead to accelerated atherosclerosis and CVD [77].

Interestingly, the importance of postprandial lipoproteins in the development of atherosclerotic vascular disease was initially proposed almost 70 years ago by Moreton who wrote “the lipid particles must be assumed to be retained and deposited from the plasma-derived nutrient lymph stream which normally passes from the lumen through the intramural structures towards the adventitial venules and lymphatics. It may be theorised that the increased particle size of the lipids in sustained or alimentary hyperlipemia is the stimulus to the phagocytosis in the intima by macrophages and the formation of the typical foam cells” [88, 89]. It is now clear that Moreton’s work has not, until now, received the attention it deserves.

### Triglyceride-Rich Lipoproteins and Remnants as Therapeutic Targets in Hypertriglyceridemia

As discussed above, recent epidemiological studies have unequivocally demonstrated that elevated levels of postprandial TG and remnant particles are clinically significant risk factors for CVD [2, 3, 6•]. Furthermore, postprandial TG concentrations have been shown to be a superior risk predictor for CVD than fasting TG [2, 3, 90]. Epidemiological data providing insight into the frequency of mild to moderate hypertriglyceridemia (approx. 150 to 800 mg/dL) in the general population as a function of age, gender and ethnicity is lacking; nonetheless, findings in the NHANES survey suggest that at least one third of the US population can be classified as hypertriglyceridemic [91]. The degree to which such hypertriglyceridemia reflects the impact of elements of dietary habits and lifestyle relative to genetic factors is indeterminate. In this context, it is however especially relevant that recent studies from several laboratories suggest that mild to moderate hypertriglyceridemia is frequently of polygenic origin, arising as a result of a cumulative burden of common and rare variants in more than 30 genes coding for proteins of the complex lipolytic system, each of these polymorphisms generating proteins with mildly attenuated biological activity [13••]. A genetic score approach is therefore meaningful. Clearly then, an emerging body of evidence supports the contention that, from a therapeutic perspective, efforts to efficaciously reduce circulating concentrations of TRLs and their remnants have become critical.

### Management of Hypertriglyceridemia and New Therapeutic Options

Following exclusion of secondary causes, treatment of mild to moderate hypertriglyceridemia should follow guideline recommendations, the initial step involving counselling on dietary habits, smoking and exercise [92]. The objective in such individuals is clearly to diminish their cardiovascular risk. In the event that pharmacotherapy is required, statins, fibrates and omega-3 fatty acids are all effective agents for reduction of TG levels, but only the use of statins is supported by a solid evidence base derived from multiple randomised control intervention trials [92].

New therapeutic options are currently under development that have been based on genetic evidence for reduced cardiovascular risk in families with phenotypes involving markedly diminished TG levels and rare causative monogenic mutations. Of the candidate proteins involved, apoC-III stands out as an elegant example. Indeed, a null mutation in human *APOC3* was discovered in 2008 in the Amish community in the USA and found to provide apparent cardioprotection [35]. Abundant data now support the working hypothesis that pharmacotherapeutic reduction in circulating apoC-III levels may represent a valid target for hypotriglyceridemic therapy and ultimately for reduction of cardiovascular risk and potentially pancreatitis. Thus, loss-of-function mutations in *APOC3* are associated with low TG levels and reduced risk of ischemic heart disease in two general population studies involving more than 75,000 participants [37]. Similar findings were made in the exome sequencing project [38].

As discussed above, apoC-III is a key factor on the surface of TRL and is a critical modulator of the lipolytic activity of lipoprotein lipase. Circulating levels of apoC-III are elevated however and of the order of 10–20 mg/dL, suggesting that hepatic production of apoC-III may be more viable as a target compared to a monoclonal antibody approach to remove apoC-III protein, allowing maintenance of low plasma levels over extended periods of time [93]. It is in this context that the development of anti-sense oligonucleotides targeted to the hepatic mRNA of apoC3 hold considerable promise, as dose-dependent reductions in TG levels of up to 80 % are attainable [94].

## Conclusion

The number of patients with hypertriglyceridemia will grow significantly over the coming years, partly due to the increase in patients with diabetes mellitus type 2 and metabolic syndrome. Indeed, hypertriglyceridemia poses a major emerging challenge for public health and requires adequate targeting. As current therapies are not optimal for normalisation of elevated TG levels, the development of novel therapeutic agents is therefore warranted.
